# Supplementary figures and images for: Learning from stakeholders to inform good practice guidance on consent to research in intensive care units: a mixed-methods study
Source: BMJ Open. 2022 Nov 14;12(11):e066149. doi: 10.1136/bmjopen-2022-066149 (PMC9664286; doi:10.1136/bmjopen-2022-066149)

## Supplementary File 1

### Perspectives CONSORT Flow Diagram

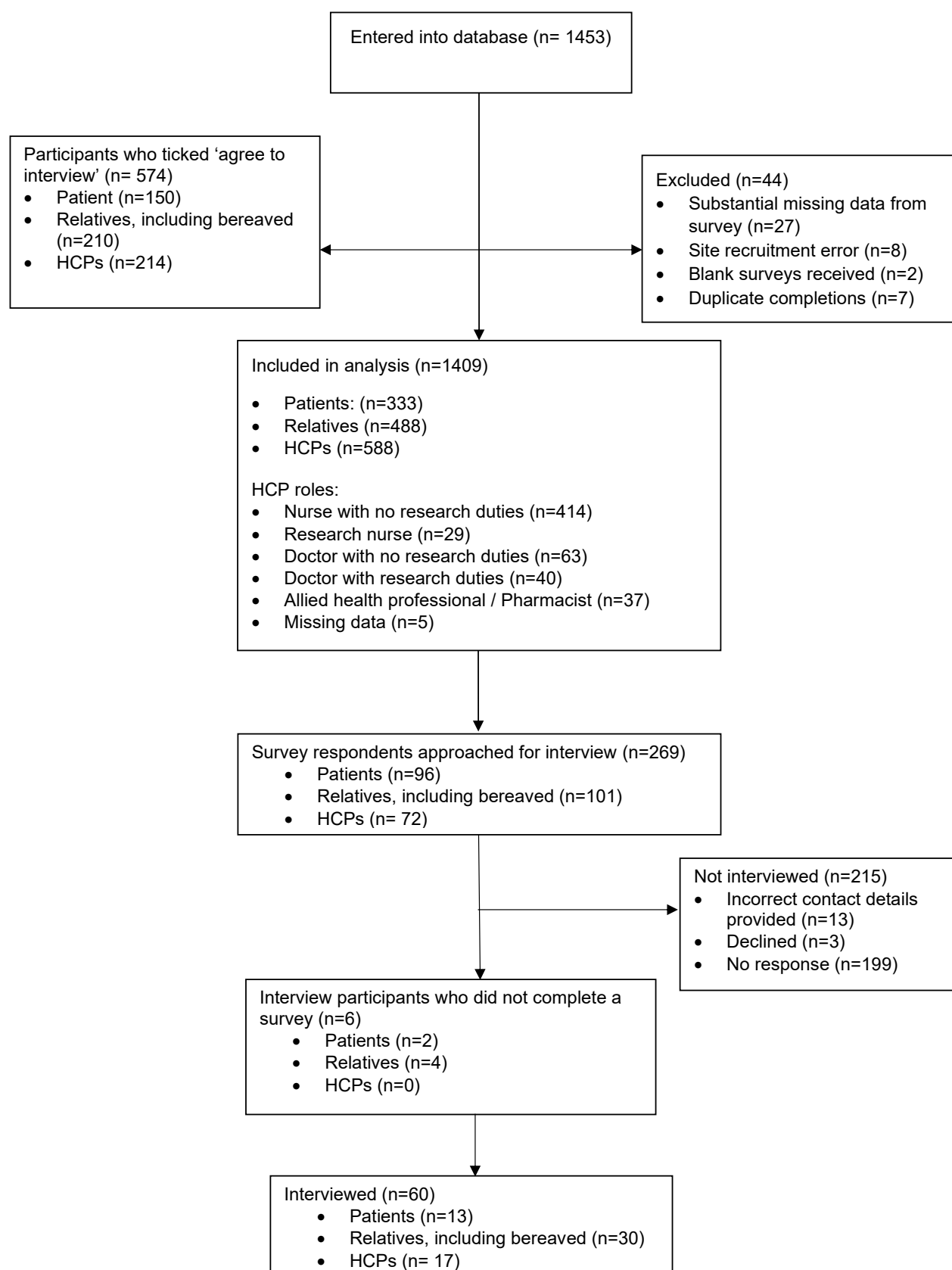

Supplement: Supplementary data [file bmjopen-2022-066149supp001.pdf]
