# Supplementary material for: Learning from stakeholders to inform good practice guidance on consent to research in intensive care units: a mixed-methods study
Source: BMJ Open. 2022 Nov 14;12(11):e066149. doi: 10.1136/bmjopen-2022-066149 (PMC9664286; doi:10.1136/bmjopen-2022-066149)
Supplement: Supplementary data [file bmjopen-2022-066149supp002.pdf]

Supplementary File 2

Table 2: Interview sample

	Category	Patients N=13 n(%)	Relatives N=30 n(%)	HCPs N=17 n(%)
**Gender**	Male	5 (39)	11 (37)	6 (35)
	Female	8 (62)	19 (63)	11 (66)
**Age**	18 to24	1 (8)	2 (7)	1 (6)
	25 to 34	2 (15)	4(13)	8 (47)
	35 to 44	1 (8)	6 (20)	4 (24)
	45 to 54	2 (15)	5 (17)	3 (18)
	55 to 64	2 (15)	5 (17)	0 (0)
	65 to 74	4 (31)	3 (10)	0 (0)
	75+	1 (8)	1 (3)	0 (0)
	Unknown	0 (0)	4 (13)	1(6)
**Relationship to patient**	Son/Daughter	N/A	12 (30)	N/A
	Brother/Sister	N/A	1 (3)	N/A
	Mother/Father	N/A	3 (10)	N/A
	Spouse/Partner	N/A	13 (43)	N/A
	Friend	N/A	0 (0)	N/A
	Other	N/A	1 (3)	N/A
**Socio-economic status***	High	3 (23)	10 (33)	N/A
	Medium	3 (23)	7 (23)	N/A
	Low	4 (31)	11 (37)	N/A
	Unknown	3 (23)	2 (7)	N/A
**Previous experience with research****	Yes	6 (46)	19 (63)	13 (76)
	No	7 (54)	11 (37)	4 (24)
**Consented to research***	Yes	6 (46)	15 (30)	N/A
	No	0 (0)	4 (13)	N/A
	Not applicable	7 (54)	11 (37)	N/A

*only applicable to patients and relatives
** approached about research (patients and relatives), involved in the consent and recruitment to research (HCPs)

Paddock K, *et al. BMJ Open* 2022; 12:e066149. doi: 10.1136/bmjopen-2022-066149
